# Ethnic-Racial Identity and Self-Esteem as Predictors of Life Satisfaction among Asian American Emerging Adults

**DOI:** 10.1007/s40615-025-02588-2

**Published:** 2025-08-07

**Authors:** Mihyun Kim, Danbi Choe

**Affiliations:** 1 University of Georgia, Athens, USA; 2 Department of Psychology, Louisiana State University, Audubon Hall, Rm #206, Baton Rouge, Louisiana, 70803 USA

**Keywords:** Ethnic-racial identity, Self-esteem, Life satisfaction, Asian American emerging adults, Structural equation modeling (SEM)

## Abstract

Asian American emerging adults encounter unique psychological challenges, including pressures from the model minority stereotype, experiences of racial discrimination, heightened risk of social isolation, and family conflicts stemming from the cultural values differences across generations. Previous research suggests that ethnic-racial identity and self-esteem serve as foundational factors in addressing these challenges and promoting life satisfaction among Asian American emerging adults. This study examined the structural relationships among ethnic-racial identity exploration, identity commitment, self-esteem, and life satisfaction, with a particular focus on the mediating role of self-esteem. Multi-group structural equation modeling was conducted to examine these associations across gender and generational groups. The study included 544 Asian American emerging adults between the ages of 18 and 24. Results revealed that exploration had a direct positive effect on life satisfaction, whereas commitment significantly predicted self-esteem. Furthermore, exploration indirectly enhanced life satisfaction through its impact on commitment and self-esteem. Interestingly, these paths differed by generational status but not by gender. However, significant mean differences in ethnic-racial identity and self-esteem were observed across gender and generational subgroups. Our findings highlighted the complementary roles of ethnic-racial identity exploration and commitment in fostering psychological well-being. In the discussion, we proposed the implications for developing tailored interventions that consider gender and generational status to better support the mental health of Asian American emerging adults.

## Introduction

Asian American emerging adults face distinct challenges compared to their peers from other ethnic and racial groups. Suicide remains the leading cause of death among this population, with high rates of depression, stress, and emotional problems, all of which threaten their psychological well-being [1–3]. They are also subject to elevated levels of racial discrimination, which exacerbate feelings of marginalization and adversely affect their psychological well-being [4–6]. The recent surge in anti-Asian hate crimes [7] has intensified these difficulties. Additionally, the pervasive model minority stereotype that imposes unrealistic expectations for academic and occupational achievement, places significant psychological pressure on Asian American emerging adults [8–10]. Ironically, this stereotype often leads to reduced attention and support for their mental health needs, thereby perpetuating struggles [11, 12].

To effectively address existing challenges and promote psychological well-being, ethnic-racial identity and self-esteem can be two foundational pillars. Ethnic-racial identity plays a central role in shaping how Asian American emerging adults adapt and respond to these obstacles [13]. However, minority status often complicates the process of exploring and affirming one’s ethnic-racial identity within broader societal contexts [8]. Furthermore, self-esteem is a key component of psychological well-being by influencing how individuals perceive themselves and their capacity to cope with adversity [14–17]. These insights suggest that fostering self-esteem in connection with a healthy ethnic-racial identity might promote life satisfaction among Asian American emerging adults.

This study explored the relationships among ethnic-racial identity, self-esteem, and life satisfaction among Asian American emerging adults using structural equation modeling (SEM). Multi-group SEM was also used to investigate whether these relationships vary by gender and immigrant generational status. This analytic approach provided critical insight into the distinctive patterns of relationships between ethnic-racial identity, self-esteem, and life satisfaction within different subgroups.

### Ethnic-Racial Identity and Life Satisfaction Among Asian American Emerging Adults

Ethnic-racial identity refers to the beliefs and attitudes individuals hold about their ethnic and racial group memberships, as well as the processes through which these beliefs and attitudes develop over time [18, 19]. Yip and colleagues [20] argued that ethnic identity and racial identity should be studied as intersecting constructs rather than as separate dimensions of social identity, because this approach better illustrates the complexity of minority experience. Recent literature increasingly use the term ethnic-racial identity as a comprehensive construct that captures both ethnic identity (e.g. shared culture, language, national origin) and racial identity (e.g., racialized experiences) [21]. Quintana [22] highlighted the importance of tailored approach of identity development for minority adolescents to reflect specific ethnic and racial contexts.

Cross and Cross [23] suggested the difficulty in clearly understanding how individuals from minoritized backgrounds personally perceive and differentiate between race and ethnicity. As such, the use of a comprehensive term like ethnic-racial identity is appropriate to capture the complex, inseparable nature of participants’ lived experience. Given our sample consisted of racially minoritized individuals, we chose the term ethnic-racial identity to reflect the interconnected nature of ethnicity and race in identity development, even though we used the ethnic identity measurement developed by Phinney and Ong [24].

Phinney [25] identified three stages of ethnic-racial identity: unexamined, exploration, and achievement. The unexamined stage is characterized by a lack of engagement with the meaning of group membership. The exploration stage involves an active search for information about the significance of group membership. The achievement stage represents a resolution of the identity search, ideally resulting in a strong commitment to and affirmation of one’s ethnic-racial group. Based on Phinney’s framework [25], two dimensions of ethnic-racial identity are commonly discussed in the literature: exploration and commitment. Research suggests that exploration generally precedes commitment in the identity development process [26]. This progression reflects a trajectory in which individuals seek to understand their heritage (exploration) before forming a stable, meaningful connection to it (commitment).

Identity development is particularly crucial for adolescents and emerging adults from immigrant backgrounds [10, 27, 28]. Ethnic-racial identity has been shown to foster self-acceptance, self-knowledge, and coherence, thereby contributing to overall well-being [13, 29–31]. However, research examining its specific impact on life satisfaction and psychological well-being among Asian American emerging adults has remained unclear. Some studies propose that ethnic-racial identity serves as a protective factor against stressors of racial discrimination. For example, cultural pride and adherence to traditional values have been linked to enhanced psychological adjustment and life satisfaction among Asian American college students [32]. In a study of 334 Asian Americans, Chae and Foley [33] found that higher levels of ethnic-racial identity were associated with enhanced quality of life and life satisfaction. Similarly, a large-scale study involving 2,109 Filipino Americans revealed that strong identification with one's ethnic group was related to fewer depressive symptoms [34].

However, the individual effects of ethnic-racial identity exploration and commitment on psychological well-being appear more nuanced. High levels of exploration were associated with increased psychological distress [35] and elevated anxiety symptoms [36]. Under conditions of economic stress, exploration did not buffer against the negative effects of racial discrimination among Asian American youth [37]. These findings suggest that exploration alone may not serve as a protective factor and might even contribute to psychological strain.

Furthermore, the specific role of ethnic-racial identity commitment in shaping psychological well-being remains underexplored in existing research. Given that ethnic-racial identity development involves two distinct phases, focusing exclusively on one dimension or using ethnic-racial identity as a general construct might obscure the role and value of each phase. A deeper understanding of the unique contributions of exploration and commitment is needed to clarify their roles in the well-being of Asian American emerging adults.

### The Role of Self-Esteem Among Asian American Emerging Adults

Self-esteem refers to an individual’s evaluation of their self-worth or a global regard for oneself [3]. It is shaped by a variety of factors, such as personality, attachment styles, ethnic-racial identity, academic and occupational achievement, and social class [14]. Specifically, a strong ethnic-racial identity can provide a solid foundation for developing a positive self-concept [13].

However, this association can be influenced by the sociocultural contexts in which individuals are situated. When ethnic group membership is socially valued [38] or when ethnicity is a central component of an individual’s self-concept [39], positive identification with one’s ethnic group tends to enhance self-esteem. Conversely, in contexts where the ethnic group is marginalized or perceived as less valued, a strong ethnic-racial identity may not translate into positive psychological outcomes [40].

Empirical research has consistently demonstrated a positive relationship between self-esteem and life satisfaction. A meta-analysis of 74 studies found that higher levels of self-esteem predict greater life satisfaction [26]. Individuals with higher self-esteem are more likely to interpret challenges positively, which in turn leads to greater life satisfaction [41, 42]. This relationship has also been confirmed among Asian American youth. For example, a study of 1,021 Asian American adolescents found that self-esteem significantly predicted higher life satisfaction [43].

Relatively few studies have examined whether self-esteem facilitate the link between ethnic-racial identity and life satisfaction. Self-esteem might serve as a mediating factor between this relationship, as it plays a critical role in how individuals interpret and overcome challenges. Therefore, the current study hypothesized that self-esteem mediated the relationship between ethnic-racial identity and life satisfaction.

### Intersectionality: Gender and Generational Status Differences

Research on ethnic-racial identity and psychological well-being has highlighted the influence of intersectional factors such as gender and generational status. In terms of gender, Asian American men and women tend to go through distinct process of ethnic-racial identity development, which result in varied psychological outcomes. For instance, Asian American female college students were more likely to adhere to traditional Asian cultural values than male students [44]. This difference may stem from greater parental expectations for women to conform and uphold family values and cultural traditions. Another study [45] involving 1,583 immigrant Asians in the U.S. showed that family conflict was more linked to mental health problems among Asian American women, especially those with a strong ethnic-racial identity. Similarly, a study of 970 Asian American college students revealed that ethnic-racial identity mediated the relationship between family ethnic-racial socialization and psychological well-being only for female students [28]. However, these studies often compared male and female participants using mean differences in single variable (e.g., life satisfaction or ethnic-racial identity) or a relationship between a pair of two variables (e.g., ethnic-racial identity and psychological well-being, or social pressure and life satisfaction). Thus, the complex interconnections among gender, ethnic-racial identity, self-esteem, and life satisfaction remain underexplored.

Generational status, another important dimension of intersectionality, plays a critical role in shaping ethnic-racial identity and its psychological outcomes among Asian American youth. A study of 301 Asian American youth indicated that first-generation immigrants were more strongly attached to traditional Asian cultural values than second or later-generation peers [2]. Similarly, first-generation parents were more likely to retain Asian cultural norms, whereas U.S.-born second-generation children may feel less compelled to adopt these values in the context of American society [46].

These generational differences are associated with the way ethnic-racial identity impacts psychological well-being. For second-generation immigrants, ethnic-racial identity facilitated integration between their heritage culture and American identity, leading to positive mental health [24]. However, their stronger association with American society might also increase vulnerability to racial discrimination, which can threaten their sense of belonging [47]. In contrast, first-generation immigrants were more likely to maintain a primary connection to their ethnic group and showed lower levels of depression, possibly due to protective factors such as stronger parental supervision, lower parent–child conflict, higher church attendance, and greater social support [46]. Since the participants in our study were emerging adults who had likely completed some degree of ethnic-racial identity development, the generational differences might have different patterns than those from prior works focused mainly on school-aged populations.

### Current Study

**Fig. 1:**
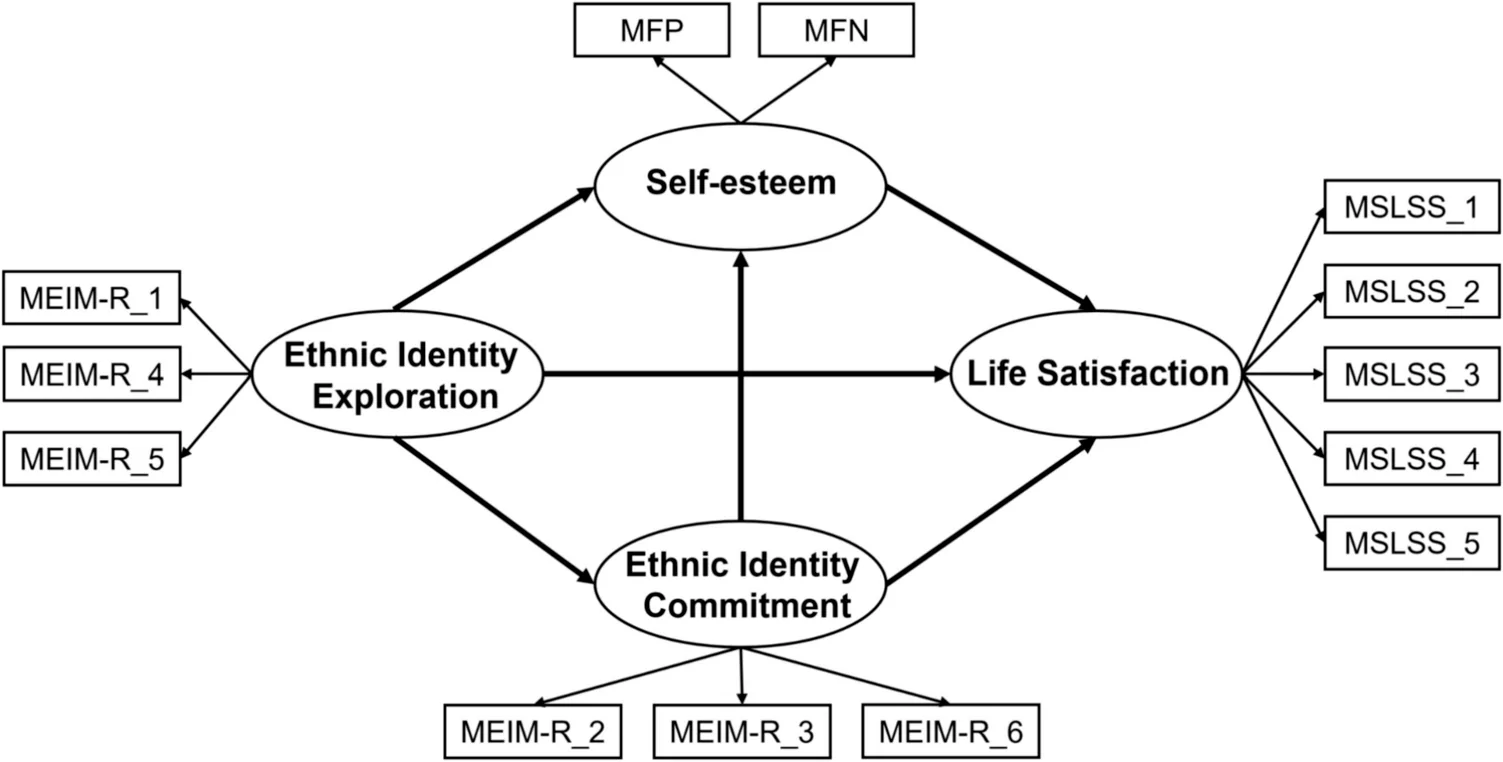
Structural model

This study aimed to investigate the structural relationships among ethnic-racial identity exploration and commitment, self-esteem, and life satisfaction in Asian American emerging adults, with a particular focus on the mediating role of self-esteem. We conceptualized ethnic-racial identity as a dynamic and developmental construct, consisted of two distinct but interconnected phases: exploration and commitment. We hypothesized a structural model in which ethnic-racial identity exploration fosters commitment, which subsequently enhances self-esteem, ultimately contributing to greater life satisfaction (see Fig. 1). We also assessed differences in the proposed model based on gender and immigrant generational status. The study addressed the following research questions (RQs):

- RQ1: What are the structural relationships among ethnic-racial identity exploration and commitment, self-esteem, and life satisfaction, and does self-esteem mediate these relationships?
- RQ2: Does the structural model among ethnic-racial identity, self-esteem, and life satisfaction differ by gender?
- RQ3: Does the structural model among ethnic-racial identity, self-esteem, and life satisfaction differ by generational status?

## Method

### Participants

Data were collected from 544 participants, comprising 49.3% women, 47.6% men, and 3.1% non-binary individuals. Participants ranged in age from 18 and 24 (M = 21.4, SD = 1.73). Of the participants, 18.5% identified as first-generation immigrants, while 81.5% identified as second-generation immigrants. The data included participants from diverse ethnic backgrounds: Chinese (29.2%), Vietnamese (15.9%), Indian (15.7%), Filipino (12.9%), Korean (7.6%), Pakistani (4.6%), Cambodian (1.1%), multiethnic (6.3%), and others ethnicities (6.1%). Specifically, multiethnic participants identified with a variety of parental backgrounds, such as Chinese-Vietnamese, Japanese-White, Filipino-Vietnamese, and Korean-White. 0.7% of participants did not disclose their ethnicity. Annual family incomes varied among participants: 19.6% earned less than $50 K, 16.6% earned between $50 K and $75 K, 18.1% earned between $75 K and $100 K, and 37.6% earned over $ 100 K. 8.1% of participants chose “Prefer Not to Say.” There were no missing data due to incomplete response, but some participants selected “Prefer not to say” for certain demographic questions. This included two for gender, five for ethnicity, and 46 for family income. Two participants with non-disclosed gender were treated as missing and handled using pairwise deletion [48]. For ethnicity and family income, these responses were coded as separate categories. The final analytic sample included 542 participants.

### Procedures

Participants were recruited from two sources: (a) Prolific, an online research recruitment platform, and (b) the university-affiliated subject pool system (SONA) at Louisiana State University in the USA. 500 participants were recruited through Prolific, and 44 participants were undergraduate students from SONA who received course credit in exchange for participation. All participants were pre-screened based on inclusion criteria, including self-identification as first- or second-generation Asian American. Although the study’s inclusion criteria allowed for individuals within the 15–24 age range, the final sample included only participants aged 18 and older. All participants provided informed consent prior to participation. This study received approval from the Institutional Review Board at Louisiana State University (IRBAM-23–0184).

### Measure

#### Ethnic-Racial Identity

Ethnic-racial identity was assessed using the Multigroup Ethnic Identity Measure-Revised (MEIM-R) [24], which includes two subscales: exploration and commitment. Each subscale includes three items rated on a 5-point Likert scale (1 = Strongly Disagree to 5 = Strongly Agree). Higher scores present greater levels of exploration of and commitment to one’s ethnic identity. This measure demonstrated strong internal reliability, with Cronbach’s α above 0.70. McDonald’s omega reliability coefficients were 0.81 for the exploration subscale and 0.87 for the commitment subscale, indicating good reliability. Previous research supported the two-factor model, showing a good fit in a sample of 1,463 young adults from diverse ethnicities [49]. In our study, CFA results demonstrated good model fit and validity (CFI = 0.995, TLI = 0.991, RMSEA = 0.043, 90% CI = [0.005, 0.074], SRMR = 0.021).

#### Self-Esteem

The Rosenberg Self-Esteem Scale (RSES) [50] is a widely used measure for assessing self-esteem. The scale includes five positively worded items (e.g., "I take a positive attitude toward myself") and five negatively worded items (e.g., "All in all, I am inclined to feel that I am a failure"). Negatively worded items were reverse-scored. Participants rated each item on a 4-point Likert scale (1 = Strongly Disagree to 4 = Strongly Agree). Previous research on the RSES [51] suggested that the scale consisted of a general self-esteem (GSE) factor and two factors of positively worded items (MFP) and negatively worded items (MFN), which capture different psychological dimensions. However, a two-factor model has been shown to provide an adequate fit across diverse cultural groups, including adolescents in Asian countries (China, α = 0.83 and Korea, α = 0.71) [16]. In this study, McDonald’s omega reliability coefficient for both the MFP and MFN subscale were 0.89, suggesting strong reliability. CFA of the two-factor model, which allowed correlations between items 3 and 4 and items 2 and 6, indicated good model fit (CFI = 0.973, TLI = 0.962, RMSEA = 0.068, 90% CI = [0.054, 0.082], SRMR = 0.037).

#### Life Satisfaction

Life satisfaction was assessed using the Brief Multidimensional Students’ Life Satisfaction Scale-College Version (BMSLSS-C) [52]. This scale includes one item for each of seven domains: family, friendships, school experiences, self-perception, living environment, romantic relationships, and physical appearance. However, CFA of the current data showed low factor loadings (< 0.40) for the romantic relationships and physical appearances items. Consistent with the scale’s original validation study [53], these two items were dropped from the final analytic model. The revised model included five core domains: family, friendships, school experiences, self-perception, and living environment. The overall life satisfaction item was retained as a validity check.

The response format was changed from the original 7-point Likert scale (1 = Terrible to 7 = Delighted) to a 5-point Likert scale (1 = Strongly Dissatisfied to 5 = Strongly Satisfied). This modification was informed by psychometric findings suggesting that response formats with fewer response categories yield higher-quality data [54]. A 5-point version of the BMSLSS-C has also been used in previous studies [55, 56]. The scale demonstrated acceptable internal reliability and criterion-related validity in a sample of 723 college students [52]. In this study, McDonald’s omega reliability coefficient for the life satisfaction scale was 0.81, indicating good reliability. CFA result showed good model fit (CFI = 0.988, TLI = 0.961, RMSEA = 0.067, 90% CI = [0.025, 0.115], SRMR = 0.020).

### Analytical Strategies

The analytic procedures involved three steps: (a) preliminary analyses, (b) SEM analysis, (c) multi-group SEM analyses based on gender (i.e., men and women) and immigrant generational status (i.e., first- and second-generation). As part of preliminary analyses, descriptive statistics and correlation coefficients for the study variables were reported in Table 1.

**Table 1:** Descriptive statistics and correlation

	①	②	③	④	⑤	⑥	⑦	⑧	⑨	⑩	⑪	⑫	⑬
①	1												
②	.63***	1											
③	.51***	.58***	1										
④	.40***	.48***	.36***	1									
⑤	.40***	.50***	.39***	.66***	1								
⑥	.47***	.53***	.41***	.74***	.63***	1							
⑦	.10*	.12***	.13***	.24***	.18***	.19***	1						
⑧	.02	.05	.07	.19***	.11*	.12*	.75***	1					
⑨	.04	.12*	.10*	.18***	.13***	.16***	.43***	.35***	1				
⑩	.05	.11*	.12*	.14***	.13***	.15***	.42***	.33***	.38***	1			
⑪	.04	.08	.10*	.13***	.13***	.12*	.47***	.44***	.33***	.48***	1		
⑫	.12*	.19***	.15***	.21***	.18***	.20***	.75***	.69***	.47***	.49***	.54***	1	
⑬	-.02	.03	.02	.12***	.08	.08	.24***	.20***	.35***	.26***	.31***	.28***	1
M	3.85	3.81	3.69	3.76	3.80	3.92	3.62	2.75	3.45	3.60	3.20	3.11	3.51
SD	0.95	0.98	1.10	1.09	0.99	1.07	0.84	0.98	1.16	1.09	1.15	1.18	1.14
SK	−0.81	−0.8	−0.66	−0.69	−0.79	−0.89	−0.74	0.26	−0.53	−0.69	−0.37	−0.32	−0.52
KT	0.02	0.17	−0.44	−0.43	0.12	0.07	0.35	−0.67	−0.73	−0.37	−0.87	−1.07	−0.57

M = mean, SD = standard deviation, SK = skewness, KT = kurtosis, ethnic identity exploration (① MEIM-R_1, ② MEIM-R_4, ③ MEIM-R_5), ethnic identity commitment (④ MEIM-R_2, ⑤ MEIM-R_3, ⑥ MEIM-R_6), self-esteem (⑦ MFP, ⑧ MFN), life satisfaction (⑨ MSLSS_1, ⑩ MSLSS_2, ⑪ MSLSS_3, ⑫ MSLSS_4, ⑬ MSLSS_5)

To examine the structural relationships among ethnic-racial identity exploration, commitment, self-esteem, and life satisfaction, SEM was conducted following a two-step approach [57]. First, CFA was performed to confirm whether the observed variables appropriately represented the latent constructs. Second, the model fit of the structural model and the significance of path coefficients were assessed. In addition to the chi-square test, which is sensitive to sample size, fit indices such as the Comparative Fit Index (CFI), Tucker-Lewis Index (TLI), and Root Mean Square Error of Approximation (RMSEA) were used to evaluate model fit. The indirect effects of ethnic-racial identity exploration and commitment on life satisfaction, mediated by self-esteem, were tested using the bootstrap method.

Multi-group SEM analyses based on gender and generational status were conducted in two phases. We first assessed the group differences in the means of four variables (i.e., ethnic-racial identity exploration, commitment, self-esteem, and life satisfaction). To ensure that these psychological constructs were measured equivalently across groups, a series of measurement invariance tests were conducted, including configural, metric, scalar, and strict invariance. Configural invariance checked whether the overall factor structure was consistent across groups. Metric invariance constrained factor loadings to determine whether the relationships between latent and observed variables were same across groups. Scalar invariance assessed whether the intercepts of items were equal across groups. Strict invariance tested whether the factor loadings, intercepts, and residual variances were equivalent across groups. Once these invariances were confirmed, observed mean differences reflected true differences in the latent variables. Effect sizes were interpreted based on Cohen's criteria [58]. After confirming measurement invariance, structural invariance was tested to determine whether path coefficients were equivalent across groups. Because structural invariance was not satisfied, the metric invariance model was set as the baseline. Constraints were applied one by one to each path to identify group differences.

All statistical analyses were performed using R version 4.3.2. [59]. Gender (i.e., men, women, non-binary), ethnicity (i.e., Chinese, Vietnamese, Indian, Filipino, Korean, Pakistanis, Cambodian, multiethnic, others), and family income (i.e., less than $50,000; $50,000—$74,999; $75,000—$99,999; $100,000 or more) were dummy coded and included as covariates. For the multi-group SEM analysis by gender, participants who identified as non-binary were excluded, resulting in a missing data rate of 3.1% (n = 17).

## Result

### Descriptive Analysis and Correlations

A descriptive analysis was conducted to test the normality assumption for 13 measured variables, including three ethnic-racial identity exploration scores, three ethnic-racial identity commitment scores, two self-esteem scores, and five life satisfaction scores (see Table 1). The absolute values of skewness for all variables were below 2, and kurtosis values were below 7, indicating that the assumption of normality was met. These results justified the use of the maximum likelihood estimation method. Pearson correlation coefficients among the measurement variables were presented in Table 1. Self-esteem showed a positive correlation with life satisfaction. Ethnic-racial identity commitment was positively and significantly correlated with both self-esteem and life satisfaction. In contrast, ethnic-racial exploration was weakly correlated with self-esteem and life satisfaction.

### Analysis of Structural Model

CFA was conducted to test the measurement model, which included four latent variables (ethnic-racial identity exploration and commitment, self-esteem, and life satisfaction) and 13 observed variables capturing those latent variables. The measurement model exhibited good fit (CFI = 0.989, TLI = 0.985, RMSEA = 0.034, 90% CI = [0.021, 0.047], SRMR = 0.033). All observed variables significantly converged onto their respective latent variables. Correlations among the latent variables ranged from 0.153 to 0.890 and did not exceed 0.9, supporting the model’s construct validity.

Based on the measurement model, a structural model examined the structural relationships among ethnic-racial identity exploration, commitment, self-esteem, and life satisfaction (see Fig. 1). The model fit was acceptable (${x}^{2}$= 208.188, *df* = 117, CFI = 0.972, TLI = 0.963, RMSEA = 0.038). Path coefficients were presented in Table 2. Ethnic-racial identity exploration had a positive relationship with commitment (*β* = 0.704). Commitment was positively related to self-esteem (*β* = 0.300). Both exploration (*β* = 0.123) and self-esteem (*β* = 0.892) were positively associated with life satisfaction. However, the paths from exploration to self-esteem (*p* = 0.382) and from commitment to life satisfaction (*p* = 0.444) were not significant.

**Table 2:** Path coefficient of structural model

Path	b	SE	*β*	t
EXP → COM	0.950	0.070	0.704	13.653***
EXP → SE	−0.073	0.084	−0.068	−0.875
EXP → LS	0.105	0.045	0.123	2.334*
COM → SE	0.240	0.062	0.300	3.885***
COM → LS	−0.026	0.033	−0.041	−0.765
SE → LS	0.705	0.062	0.892	11.392***

EXP = ethnic identity exploration, COM = ethnic identity commitment, SE = self-esteem, LS = life satisfaction, b = unstandardized coefficient*,* SE = standard error, *β* = standardized coefficient*;* *p < .05, **p < .01, ***p < .001

To examine the indirect effects of ethnic-racial identity on life satisfaction mediated by self-esteem, a bootstrap analysis (n = 5,000) was conducted (see Table 3). A significant direct effect was found from ethnic-racial identity exploration to life satisfaction (EXP → LS; *β* = 0.123). An indirect effect from exploration to commitment to self-esteem to life satisfaction was significant (EXP → COM → SE → LS; *β* = 0.187). This indirect effect suggested that exploration promoted commitment, which in turn enhanced self-esteem, ultimately leading to greater life satisfaction. However, the indirect effects along the paths EXP → SE → LS (*p* = 0.669) and EXP → COM → LS (*p* = 0.677) were not significant. The total effect of exploration on life satisfaction was significant (*β* = 0.236), indicating a meaningful cumulative impact of direct and indirect paths.

**Table 3:** Mediation effect analysis

Path	Direct effect		Indirect effect		Total effect	
	b	*β*	b	*β*	b	*β*
EXP → LS	0.105	0.123*			0.208	0.236***
EXP → SE → LS			−0.030	−0.034		
EXP → COM → LS			−0.017	−0.019		
EXP → COM → SE → LS			0.165	0.187**		

EXP = ethnic identity exploration, COM = ethnic identity commitment, SE = self-esteem, LS = life satisfaction, b = unstandardized coefficient*,* SE = standard error, *β* = standardized coefficient*;* *p < .05, **p < .01, ***p < .001

### Analysis of Latent Mean and Path Differences by Gender

The multi-group analysis examined the moderating effects of gender within the structural model (See Table 4). First, the sample was divided into two groups based on gender: men and women. Participants who identified as non-binary (3.1%, n = 17) were excluded from this analysis. The validity of the measurement model for both groups was confirmed (men: ${x}^{2}$ = 227.213, *df* = 177, CFI = 0.967, TLI = 0.956, RMSEA = 0.033; women: ${x}^{2}$ = 232.388, *df* = 177, CFI = 0.968, TLI = 0.957, RMSEA = 0.034).

**Table 4:** Invariance test of gender model

Model	${x}^{2}$	*df*	CFI	RMSEA	Δ ${x}^{2}$	Δ*df*	Δ CFI
Total sample	283.54	177	0.967	0.034	-	-	-
Men	227.21	177	0.967	0.033	-	-	-
Women	232.39	177	0.968	0.034	-	-	-
Configural invariance	459.60	354	0.967	0.034	-	-	-
Metric invariance	471.91	363	0.966	0.034	12.31	9	0.001
Scalar invariance	497.92	372	0.961	0.036	26.01**	9	0.005
Strict invariance	517.52	385	0.959	0.036	19.60	13	0.002
Structural invariance	460.39	360	0.969	0.033	0.79	6	0.002

**p < .01

Second, a series of measurement invariance across groups were tested. Configural invariance was confirmed (${x}^{2}$ = 459.60, *df* = 354, CFI = 0.967, and RMSEA = 0.034), indicating that the factor structure was equivalent across groups. Metric invariance found to be supported. Because the ${x}^{2}$ difference between the metric and configural invariance model was not statistically significant (*p* = 0.20), and the difference in CFI was 0.001, remained below the cutoff of 0.01 [60]. Scalar invariance was also met. Although the ${x}^{2}$ difference was statistically significant at the *p* = 0.01 level, the difference in CFI was 0.005 [60]. Finally, strict invariance was achieved, as the CFI difference between the scalar and strict invariance was 0.002. It suggested that common standard deviations could be used for comparing latent means.

Given all measurement invariance tests were satisfied, latent mean comparison were conducted using the men’s group as the reference. As shown in Table 5**,** women had significantly higher latent means for ethnic-racial identity exploration (Δ mean = 0.50, *d* = −0.24), and lower latent means for self-esteem (Δ mean = −0.35, *d* = 0.24) compared to men. Based on Cohen's effect size (*d*), there were medium effect sizes of group differences in exploration and self-esteem among Asian American emerging adults.

**Table 5:** Latent mean difference by gender

	Latent mean difference	Cohen’s d	Effect size
Ethnic identity exploration	0.50**	−0.24	medium
Ethnic identity commitment	−0.09	0.04	-
Self-esteem	−0.35**	0.24	medium
Life satisfaction	−0.50	0.16	small

Men group is criterion group; Latent mean differences are calculated by subtracting the scores of the men group from those of the women group; 2 = small, .5 = moderate, .8 = large effect size; **p < .01

To analyze the differences in path coefficients by gender, we confirmed that configural and metric invariance showed no significant difference (see Table 4). There were no significant group differences in the paths between men and women (see Table 6).

**Table 6:** Path difference test of gender group model

Model	${x}^{2}$	*df*	CFI	RMSEA	Δ ${x}^{2}$	Δ*df*	Δ CFI
Configural invariance	459.60	354	0.967	0.034	-	-	-
Metric invariance	471.91	363	0.966	0.034	12.31	9	0.001
EXP → COM	459.76	355	0.967	0.033	12.15	8	0.001
EXP → SE	459.68	355	0.966	0.034	12.23	8	0.000
EXP → LS	459.69	355	0.966	0.034	12.22	8	0.000
COM → SE	459.61	355	0.966	0.034	12.30	8	0.000
COM → LS	459.60	355	0.966	0.034	12.31	8	0.000
SE → LS	459.66	355	0.966	0.034	12.25	8	0.000

EXP = ethnic identity exploration, COM = ethnic identity commitment, SE = self-esteem, LS = life satisfaction; each path was compared with the model fit of metric invariance

### Analysis of Latent Mean and Path Differences by Generational Status

Following the same analytic procedure used in the gender-based SEM analysis, the moderating effects of immigration generational status were examined (See Table 7). The sample was divided into two groups: first- generation and second-generation immigrants. The validity of the measurement model for both groups was valid (first-generation: ${x}^{2}$ = 192.980, *df* = 177, CFI = 0.977, TLI = 0.970, RMSEA = 0.031; second-generation: ${x}^{2}$ = 288.236, *df* = 177, CFI = 0.956, TLI = 0.942, RMSEA = 0.038).

**Table 7:** Invariance test of generation group model

Model	${x}^{2}$	*df*	CFI	RMSEA	Δ ${x}^{2}$	Δ*df*	Δ CFI
Total sample	307.877	187	0.962	0.035	-	-	-
First-generation	192.980	177	0.977	0.031	-	-	-
Second-generation	288.236	177	0.956	0.038	-	-	-
Configural invariance	481.22	354	0.960	0.037	-	-	-
Metric invariance	487.75	363	0.961	0.036	6.53	9	0.001
Scalar invariance	494.93	372	0.962	0.035	7.18	9	0.001
Strict invariance	517.95	385	0.959	0.036	23.02*	13	0.003
Structural invariance	484.57	360	0.961	0.036	3.35	6	0.001

*p < .05

Next, configural invariance was achieved, indicating that the overall factor structures was equivalent across groups (${x}^{2}$ = 481.22, *df* = 354, CFI = 0.960, RMSEA = 0.037). Metric invariance found to be assumed, as the ${x}^{2}$ difference between the metric and configural models was not statistically significant (*p* = 0.69), and the difference in CFI was 0.001. Scalar invariance was confirmed, with no significant difference between the scalar and metric models (*p* = 0.62). Strict invariance was achieved, as the difference in CFI was 0.001, despite the ${x}^{2}$ difference was statistically significant. These results confirmed that standard deviations could be used to compare latent means across groups.

Given all assumptions were satisfied, latent mean comparisons were conducted using the first-generation group as the reference. As shown in Table 8, the second-generation group had significantly higher latent means for ethnic-racial identity exploration (Δ mean = 0.65) and commitment (Δ mean = 0.69), but lower means for self-esteem (Δ mean = −0.49). These differences exhibited small effect sizes, based on Cohen's effect size (*d*). In conclusion, generational status was related to meaningful latent mean differences in ethnic-racial identity and self-esteem among Asian American emerging adults.

**Table 8:** Latent mean difference by generational status

	Latent mean difference	Cohen’s d	Effect size
Ethnic identity exploration	0.65**	−0.31	Small
Ethnic identity commitment	0.69*	−0.28	Small
Self-esteem	−0.49**	0.33	Small
Life satisfaction	−0.47	0.15	Small

First-generation group is criterion group; *p < .05, **p < .01, ***p < .001

To analyze group differences in path coefficients, configural and metric invariance were confirmed (see Table 9). There were significant differences in path coefficients between the two groups (see Table 10). Both groups showed similar patterns in the following paths: Ethnic-racial identity exploration was positively associated with commitment. This path was slightly more substantial for the second-generation (*β* = 0.741) than for the first-generation (*β* = 0.644). Self-esteem had positive relationship with life satisfaction in both groups (first-generation, *β* = 0.881; second-generation, *β* = 0.898). We found no significant direct effect of exploration on self-esteem (first-generation, *p* = 0.94; second-generation, *p* = 0.87) and commitment on life satisfaction (first-generation, *p* = 0.88; second-generation, *p* = 0.35). Notably, certain paths were significant only in the second-generation group. Specifically, exploration had a significant direct effect on life satisfaction (*β* = 0.137), and commitment significantly predicted self-esteem (*β* = 0.295*).*

**Table 9:** Path Difference test of generation group model

Model	${x}^{2}$	*df*	CFI	RMSEA	Δ ${x}^{2}$	Δ*df*	Δ CFI
Configural invariance	481.22	354	0.960	0.037	-	-	-
Metric invariance	487.75	363	0.961	0.036	6.53	9	0.001
EXP → COM	376.42	275	0.968	0.037	111.33*	88	0.007
EXP → SE	376.07	275	0.968	0.037	111.68*	88	0.007
EXP → LS	377.13	275	0.968	0.038	110.62	88	0.007
COM → SE	376.35	275	0.968	0.037	111.40*	88	0.007
COM → LS	376.12	275	0.968	0.037	111.63*	88	0.007
SE → LS	376.57	275	0.968	0.037	111.18*	88	0.007

EXP = ethnic identity exploration, COM = ethnic identity commitment, SE = self-esteem, LS = life satisfaction; each path was compared with the model fit of metric invariance; *p < .05

**Table 10:** Path coefficient difference by generational status

	First-generation		Second-generation	
	b	*β*	b	*β*
EXP → COM	0.893	0.644***	0.997	0.741***
EXP → SE	0.011	0.013	−0.018	−0.016
EXP → LS	−0.003	−0.004	0.123	0.137*
COM → SE	0.171	0.267	0.244	0.295**
COM → LS	0.007	0.016	−0.041	−0.062
SE → LS	0.588	0.881***	0.725	0.898***

EXP = ethnic identity exploration, COM = ethnic identity commitment, SE = self-esteem, LS = life satisfaction; b = unstandardized path coefficient*, β* = unstandardized path coefficient*;* *p < .05, **p < .01, ***p < .001

## Discussion

This study examines the structural relationships among ethnic-racial identity exploration, commitment, self-esteem, and life satisfaction in Asian American emerging adults, with a particular focus on the mediating role of self-esteem. We also investigate whether these relationships differed by gender and generational status.

In response to research question #1, which examines the structural relationship among ethnic-racial identity exploration, commitment, self-esteem, and life satisfaction, as well as the mediating role of self-esteem, our findings reveal a set of complex patterns. First, exploration predicts the engagement in the commitment, reflecting the theoretical foundation of a dynamic and sequential process of ethnic-racial identity development [25]. This result aligns with previous research suggesting that a deeper understanding of one’s ethnic-racial identity often precedes and fosters one’s sense of commitment to their group [61–63].

Importantly, our findings highlight the distinct yet complementary roles of ethnic-racial identity exploration and commitment in shaping self-esteem and life satisfaction. Specifically, ethnic-racial identity exploration is positively associated with life satisfaction, but not with self-esteem. Conversely, ethnic-racial identity commitment significantly predicts self-esteem but has no direct effect on life satisfaction. This suggests that questioning and discovering one’s ethnic-racial background may enrich one’s sense of purpose and meaning in life, even if it does not immediately enhance self-esteem. In contrast, having a stable sense of ethnic-racial identity may contribute more directly to how individuals value themselves. These findings support earlier studies suggesting that ethnic-racial identity is positively associated with psychological well-being [13, 29–34]. By distinguishing exploration from commitment, this study advances literature by clarifying their unique and complementary roles in promoting psychological well-being among Asian American emerging adults.

The strong association between self-esteem and life satisfaction echoes past research [26, 41–43, 64]. Our results extend this body of work by demonstrating that self-esteem not only directly predicts life satisfaction but also mediates the relationship between ethnic-racial identity commitment and life satisfaction. This finding implies that, for Asian American emerging adults, an active exploration and a deeper commitment to their ethnic-racial identity foster a sense of pride, which in turn enhance life satisfaction. Therefore, interventions should not only focus on promoting identity development but also strengthening personal worth and self-acceptance.

Regarding research question #2, our findings reveal significant gender differences in the mean levels of key variables, but not in the path coefficients. Specifically, women report higher levels of ethnic-racial identity exploration and lower self-esteem compared to men. These results are consistent with the conclusions drawn by prior studies [2, 43, 44]. However, the structural paths of the model remain the same across gender groups. These findings contribute to the limited body of research on gendered experiences of ethnic-racial identity and its impact on psychological well-being, particularly among Asian American emerging adults. Our findings suggest that interventions should be adapted based on gender-specific patterns in identity development and psychological well-being. For example, intervention studies tailored for Black/African American girls [65] and boys [66] addressed unique identity empowerment strategies by gender. Given that Asian American men demonstrate lower levels of ethnic-racial identity exploration, they might benefit from mentorship programs that connect them with role models who share personal experiences about navigating their ethnic-racial identity [67]. For Asian American women, who report higher levels of exploration but lower self-esteem, may engage in interventions focused on building self-worth, such as self-affirmation writing activities [68].

Addressing research question #3, our study uncovers differences in both the mean levels of key variables and the structural relationships within the model by generational status. Regarding the mean differences, second-generation immigrants reports higher levels of ethnic-racial identity exploration and commitment compared to first-generation immigrants. This finding contrasts with earlier studies suggesting that first-generation immigrants tend to more deeply engage in their ethnic-racial identity [2, 69].

One possible explanation for this discrepancy lies in the distinct contexts each group are situated. For first-generation immigrants, ethnic-racial identity may feel more ingrained and tied to their daily lives, reducing the need for active exploration or deliberate commitment [24]. Their ethnic-racial identity might be feel assumed and natural, requiring less conscious affirmation [70]. In contrast, second-generation immigrants grow up in a bicultural environment where they might face greater identity-related conflicts and more difficulties in reconciling their heritage with the U.S. culture [46, 71]. These surroundings might necessitate intentional engagement in identity development, leading to higher levels of self-perceived exploration and commitment. Another possible explanation is that, as second-generation immigrants have spent more time in the U.S., they may have more opportunities to reflect on their identity in the U.S. cultural settings and integrate their heritage culture into their social experiences. This experience might assist in understanding the personal and societal meaning of ethnic-racial identity in the U.S. context.

In line with previous research [46, 47, 71, 72], first-generation immigrants in our study exhibit higher self-esteem than second-generation immigrants. This finding could be attributed to their greater accessibility to protective factors such as stronger parental supervision, lower levels of parent–child conflict, higher church attendance, and more extensive social support [2, 3, 46]. Interestingly, despite second-generation immigrants demonstrating higher levels of ethnic-racial identity exploration and commitment, which are factors generally associated with greater self-esteem, this group reports lower self-esteem in our study. This counteractive finding suggests that self-esteem is a complex psychological construct influenced by various individual and social factors.

Significant differences in the structural model are observed between the two generational groups. The paths from exploration to commitment and from self-esteem to life satisfaction are significant for both groups, with slightly stronger associations observed among second-generation participants. Notably, the path from commitment to self-esteem is significant only in the second-generation group. This finding indicates that for first-generation groups, commitment to ethnic-racial group may not actively translate into a source of their self-esteem.

These generational differences highlight the need for tailored interventions. For second-generation immigrants, a structured intervention utilizing the serial paths (e.g., starting with exploration, leading to enhanced commitment and self-esteem, and ultimately promoting life satisfaction) could be particularly effective. Even though this study did not evaluate specific ethnic-racial identity enhancement activities, the findings could inform a conceptual foundation for developing an intervention that consists of four phases: (a) Exploration (e.g., creating a visual timeline that highlights culturally significant moments, such as learning heritage language, experiencing discrimination, or attending a cultural event); (b) Commitment (e.g., based on the timeline, identifying what moment gives them the most pride and articulating what being Asian American means to them now); (c) Self-esteem (e.g., sharing affirmations and cultural strengths discovered in the previous phases with peers in a supportive setting); and (d) Life satisfaction (e.g., setting future goals, identifying cultural values they wish to pass down, and envisioning contributions to the community as Asian Americans). This approach could help build a strong, healthy ethnic-racial identity that successfully transmits into positive psychological outcomes.

For first-generation immigrants, given the weaker link between ethnic-racial identity commitment and self-esteem, it is necessary to explore how other factors (e.g., parental support, cultural pride, or resilience) play a role in shaping their psychological well-being. Having ethnic-racial conversations with parents has been found to foster resilience and psychological adjustment of minoritized youth [73]. Additionally, culturally responsive small-group counseling may assist handle acculturation stress and rebuild sources of self-worth for first-generation Asian American emerging adults.

### Limitations and Future Research

Although this study explored the structural relationship among ethnic-racial identity exploration and commitment, self-esteem, and life satisfaction and provided nuanced insights into these relationships across gender and generational status, several limitations should be acknowledged. First, the study was based on self-reported data, which may lead to response biases and limit the accurate reflection of experiences or feelings. Future research would benefit from incorporating multiple data sources. Second, although gender, ethnicity, and socioeconomic status were included as covariates, the model did not account for broader ecological factors. As ethnic-racial identity and psychological outcomes could be influenced by various contextual factors such as peer relationships, neighborhood characteristics, and school environments, future research is recommended to incorporate these ecological factors. Third, while the sample included diverse Asian American ethnic groups, we did not illustrate the unique differences across ethnic groups. Notably, 6% of participants identified as multiethnic. This subgroup might engage in more complex identity development processes. Future studies could investigate how intersecting ethnic backgrounds influence ethnic-racial identity and psychological well-being. Although the sample included participants from diverse ethnic backgrounds and recruitment sources, it might overrepresent individuals who were more interested in identity-related research. This selection procedure may limit the generalizability of the findings to the broader population of Asian American emerging adults. Lastly, the sample included a higher proportion of second-generation participants compared to first-generation participants. Although statistical tests of model equivalence were conducted, this skewed sample may limit the generalizability of findings.

## Data Availability

The data supporting the findings of this study are not publicly available due to institutional restrictions, but can be obtained from the corresponding author upon reasonable request.

## Code Availability

The code used during the current study is available from the corresponding author upon reasonable request.
